# *Shh* and ZRS enhancer colocalisation is specific to the zone of polarising activity

**DOI:** 10.1242/dev.139188

**Published:** 2016-08-15

**Authors:** Iain Williamson, Laura A. Lettice, Robert E. Hill, Wendy A. Bickmore

**Affiliations:** MRC Human Genetics Unit, MRC Institute of Genetics and Molecular Medicine, Crewe Road, Edinburgh EH4 2XU, UK

**Keywords:** 5C, Chromosome loop, Enhancer, Limb development, Super-resolution microscopy

## Abstract

Limb-specific *Shh* expression is regulated by the (∼1 Mb distant) ZRS enhancer. In the mouse, limb bud-restricted spatiotemporal *Shh* expression occurs from ∼E10 to E11.5 at the distal posterior margin and is essential for correct autopod formation. Here, we have analysed the higher-order chromatin conformation of *Shh* in expressing and non-expressing tissues, both by fluorescence *in situ* hybridisation (FISH) and by chromosome conformation capture (5C). Conventional and super-resolution light microscopy identified significantly elevated frequencies of *Shh*/ZRS colocalisation only in the *Shh*-expressing regions of the limb bud, in a conformation consistent with enhancer-promoter loop formation. However, in all tissues and at all developmental stages analysed, *Shh*-ZRS spatial distances were still consistently shorter than those to a neural enhancer located between *Shh* and ZRS in the genome. 5C identified a topologically associating domain (TAD) over the *Shh*/ZRS genomic region and enriched interactions between *Shh* and ZRS throughout E11.5 embryos. *Shh*/ZRS colocalisation, therefore, correlates with the spatiotemporal domain of limb bud-specific *Shh* expression, but close *Shh* and ZRS proximity in the nucleus occurs regardless of whether the gene or enhancer is active. We suggest that this constrained chromatin configuration optimises the opportunity for the active enhancer to locate and instigate the expression of *Shh*.

## Abstract (precis)

**Summary:** Super-resolution microscopy reveals that, during mouse limb development, enhancer-driven gene expression results in the juxtaposition of *Shh* and its limb bud-specific enhancer only within cells of the distal posterior limb bud.

## INTRODUCTION

Chromatin looping is a popular model describing how very long-range enhancers can communicate with their target gene promoter (Benabdallah and Bickmore, 2015); however, the relationship of loop formation and gene activation remains unclear. It has been suggested that enhancer-target gene contacts are preformed and present in tissues even where the target gene is not activated (Montavon et al., 2011; Ghavi-Helm et al., 2014). However, other reports indicate that enhancer-gene looping is spatially and temporally restricted to cells where the target gene is active. This includes in the developing mouse limb, where elevated levels of colocalisation of the global control region (GCR) and its target 5′ Hoxd genes is only seen in the cells of the distal posterior portion of the embryonic day (E) 10.5 limb bud (Williamson et al., 2012).

**Fig. 1.**
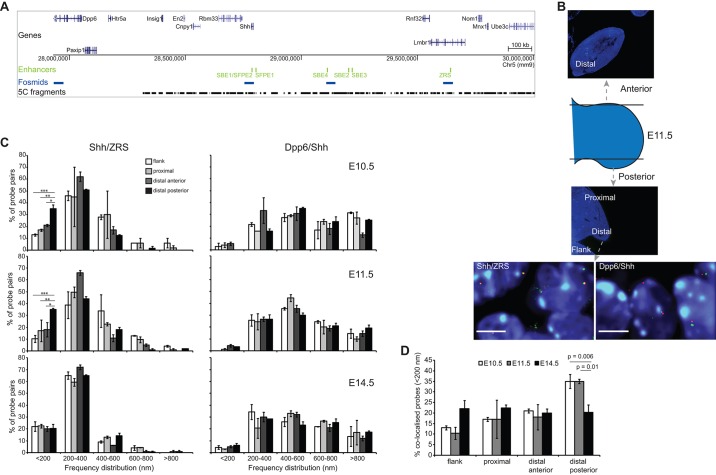
**ZRS-*Shh* proximity in the ZPA at E10.5 and E11.5.** (A) Location of genes over a 2 Mb murine genomic locus containing *Shh*, with the position of tissue-specific *Shh* enhancers shown below in green. The bottom two tracks show the locations to which the fosmid probes used for FISH hybridise (blue) and the 3C fragments amplified for 5C (black). (B) Schematic indicating the position and plane of the tissue sections taken through the anterior and posterior parts of the E11.5 forelimb bud. Distal and proximal parts of the posterior limb bud and the distal anterior limb bud are shown, as is the flank mesoderm. Below are images of nuclei from E11.5 ZPA tissue sections showing *Shh*/ZRS and *Shh*/*Dpp6* probe pairs. Scale bars: 5μm. (C) Frequency distributions of FISH inter-probe distances (d) in 200 nm bins, between *Shh* and ZRS (left column), or *Shh* and *Dpp6* probes (right column) in proximal and distal (anterior and posterior) regions of the murine forelimb bud and adjacent flank at E10.5, E11.5 and E14.5 (*n=*70-130 alleles). For E10.5 and E11.5 sections, distal posterior limb is the ZPA. Error bars represent s.e.m. obtained from two or three different tissue sections from one or two embryos. The statistical significance between data sets was examined by Fisher's exact tests: *P<0.05, **P<0.01, ***P<0.001. (D) Comparison of the proportion of colocalised *Shh*/ZRS probe pairs (<200 nm) across the three temporal developmental stages for distal anterior and posterior and proximal forelimb tissue and flank tissue. Error bars represent s.e.m. obtained from two or three different tissue sections. The statistical significance between data sets was examined by Fisher's exact tests.

The complex spatiotemporal gene regulatory circuit in the developing limb is a rich system in which to study the activity of distal regulatory elements and their mechanisms of action. The sonic hedgehog gene (*Shh*) encodes a morphogen that directs cell fate during organogenesis. Limb-specific expression of *Shh* is regulated by the ZRS enhancer positioned within an intron of *Lmbr1* ∼1 Mb away at the opposite end of a large gene desert (Lettice et al., 2002, 2003) (Fig. 1A). The ZRS has a functional role in directing spatiotemporal *Shh* expression restricted to a region of the distal posterior mesenchyme of the limb bud known as the zone of polarising activity (ZPA) (Riddle et al., 1993). Limb-specific *Shh* expression is abrogated upon deletion of ZRS (Sagai et al., 2005), whereas point mutations across the 780-bp conserved sequence of the enhancer can induce anterior, ectopic *Shh* expression and can cause preaxial polydactyly (Lettice et al., 2003, 2008; Sagai et al., 2004), triphalangeal thumb (Furniss et al., 2008) or Werner mesomelic syndrome (VanderMeer et al., 2014). Duplications, and even triplication, of the ZRS have been associated with severe forms of polysyndactyly: triphalangeal thumb-polysyndactyly syndrome and Haas type (syndactyly type IV) polysyndactyly (Klopocki et al., 2008; Sun et al., 2008; Wieczorek et al., 2010).

Previously, fluorescence *in situ* hybridisation (FISH) and chromosome conformation capture (3C) (Amano et al., 2009) have been used to demonstrate increased associations between *Shh* and ZRS in E10.5 limb buds compared with other tissues. However, no significant difference in gene/enhancer colocalisation was detected between the ZPA and distal anterior tissue, where *Shh* is not normally expressed, or indeed in ZPA cells between wild-type and embryos with a deletion of the ZRS. This would be consistent with a model of pre-formed enhancer-gene contacts. By contrast, FISH has revealed a significant decrease in *Shh*/ZRS colocalisation in E11.5 ZPA tissue from mouse embryos with a ZRS mutation that decreases ZRS long-range activity (Lettice et al., 2014), suggesting that juxtaposition of ZRS and *Shh* is directly linked to *Shh* activation.

We have previously combined FISH and 3C carbon copy (5C) to elucidate the role of chromatin conformation in the long-range regulation of the 5′ Hoxd genes during distal limb bud development (Williamson et al., 2012, 2014). Here, we combined these methods to characterise the *Shh* locus in tissue sections, including those derived from three discrete developmental stages of mouse limb bud development. Spatial proximity of *Shh* and ZRS, as inferred indirectly from enriched 5C interactions, was identified throughout E11.5 embryos, and 5C data confirmed that *Shh* and its known enhancers form a compact regulatory chromatin domain. However, using super-resolution microscopy we show that, despite *Shh* and ZRS being proximal to one another in the nucleus in all tissue types and temporal stages analysed, high levels of *Shh*/ZRS colocalisation occurs only in ZPA cells at the time of *Shh* activation. Comparison between *Shh*/ZRS distances and those between either *Shh* or ZRS and an intervening genomic locus are consistent with the formation of a chromatin loop between the active gene and enhancer.

## RESULTS

### Increased colocalisation of ZRS with *Shh* in the limb ZPA at E10.5 and E11.5

Previous analyses of the chromatin dynamics involved in the long-range regulation of *Shh* by ZRS have produced contradictory results, which could be due to the different temporal stages of development assayed (Amano et al., 2009; Lettice et al., 2014). To resolve this issue, we carried out FISH on whole mouse embryo sections that include posterior and anterior forelimb tissue from E10.5, E11.5 and E14.5 developmental stages (Fig. 1B). *Shh* is expressed within the ZPA at the two earlier stages but is switched off in the limb by E14.5 (Riddle et al., 1993). We compared inter-probe distances (Fig. S1A shows representative images) and colocalisation frequencies (Fig. 1C; left) between the gene and enhancer in mesenchymal tissue across the anterior-posterior axis of the distal forelimb bud. In addition, proximal limb tissue and the adjacent flank (where *Shh* is not expressed) were compared.

By conventional wide-field deconvolution microscopy, the proportion of colocalised (<200 nm apart) *Shh* and ZRS probe pairs in ZPA cells at stages when *Shh* is expressed (E10.5, E11.5) was significantly higher (35%) than in inactive limb regions and the flank (distal anterior *P*<0.05, proximal *P*<0.01, flank *P*<0.001; Fig. 1C; Table S2). By E14.5, there is no *Shh* expression in the limb (expression ends between E11.5 and E12.5), and the *Shh*/ZRS colocalisation frequency in distal posterior cells is significantly reduced, compared with E10.5 and E11.5 ZPA (E10.5: *P*=0.006; E11.5: *P*=0.01) (Fig. 1D). At this later stage, differences in colocalisation frequencies between the distal posterior forelimb region (∼20%) and the other limb regions and the flank mesoderm are also no longer detected (Fig. 1C).

*Dpp6* is located the same linear genomic distance away from *Shh* as the ZRS, but in the other direction and outside of the *Shh* regulatory domain (Fig. 1A). In contrast to the spatial proximity of *Shh* and ZRS, *Shh* and *Dpp6* are predominantly located >400 nm apart (colocalisation frequency <5%) (Fig. 1C; right) for all tissues and developmental stages examined.

The greater colocalisation of the active enhancer (ZRS) with its target gene (*Shh*) in the ZPA at E10.5 is similar to what we reported for these loci at E11.5 (Lettice et al., 2014) and to the colocalisation frequency of *Hoxd13* and its GCR enhancer in distal posterior expressing limb tissue and cell lines at E10.5 (Williamson et al., 2012, 2014). These differences in three-dimensional chromatin conformation between active and inactive tissues contradicts the previous report suggesting an equivalent rate of *Shh*/ZRS colocalisation on both sides of the distal limb field at this developmental stage (Amano et al., 2009).

### Super-resolution imaging identifies *Shh*/ZRS colocalisation of most alleles in the ZPA

**Fig. 2.**
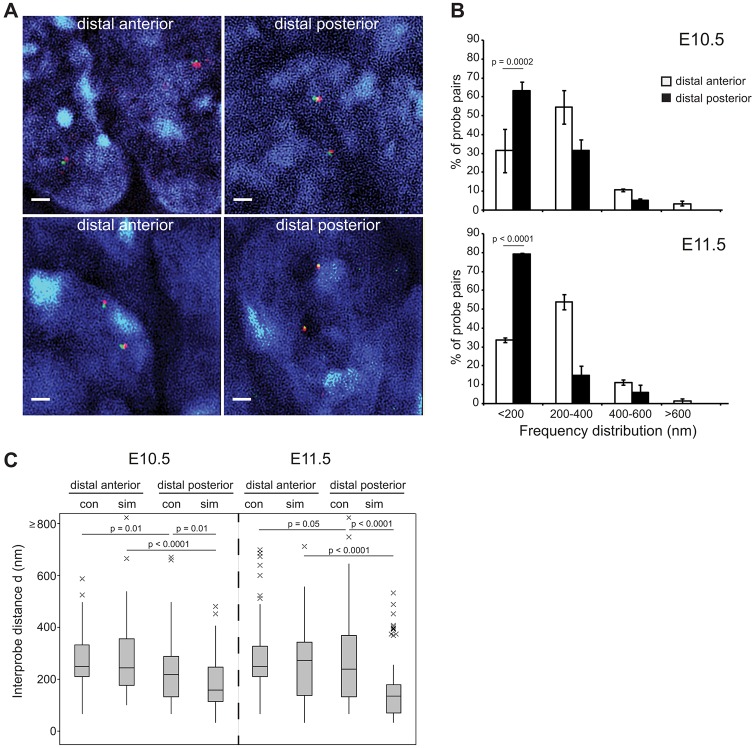
**Super-resolution imaging identifies the majority of *Shh*-ZRS probes as colocalised in ZPA tissue.** (A) Nuclei captured by super-resolution SIM imaging from the distal forelimb of E10.5 (top row) and E11.5 (bottom row) embryos after FISH with *Shh* and ZRS probe pairs. Scale bars: 1 μm. (B) Frequency distributions of *Shh*-ZRS inter-probe distances (d) measured from SIM images in 200 nm bins, in distal anterior and distal posterior regions of the murine forelimb at E10.5 and E11.5. *n=*67-100 (alleles). Error bars represent s.e.m. obtained from two different tissue sections from one embryo. The statistical significance between data sets was examined by Fisher's exact tests. (C) Boxplots show the distribution of *Shh*-ZRS inter-probe distances (d in nm) in E10.5 and E11.5 distal anterior and distal posterior limb tissue captured by conventional (con) and structured illumination (sim) microscopy. Line, median; box, interquartile range; whiskers, 95% range. The statistical significance between data sets was examined by Mann–Whitney U tests.

The data in Fig. 1 are consistent with active gene-enhancer colocalisation during long-range regulation. From the images of tissue sections from the three developmental stages, acquired by conventional light-microscopy, it was apparent that *Shh* and ZRS are consistently very close in the nucleus, with differences in spatial distance frequently down to the signal centroids being in different layers of the *z*-stack – the dimension with the lowest spatial resolution in the microscope. We therefore re-analysed the tissue sections containing E10.5 and E11.5 distal anterior and posterior (ZPA) cells by structured illumination microscopy (3D-SIM) (Fig. 2A). This technique doubles the resolution limit in all dimensions (Toomre and Bewersdorf, 2010) and has previously been combined with 3D-FISH (Nora et al., 2012; Patel et al., 2013).

The greater resolution afforded by 3D-SIM, particularly for the *z* (depth) dimension (120 nm compared with 200 nm in conventional wide-field microscopy), not only confirmed the difference in *Shh*/ZRS colocalisation frequency between ZPA and distal anterior limb bud but also suggests that conventional microscopy does not fully capture the proportion of colocalised *Shh*/ZRS probe pairs, especially in the *Shh*-expressing tissues where it now peaks at 79% (Fig. 2B; Table S3). These data suggest that a substantial proportion of *Shh*/ZRS probe pairs with signal centroids not in the same plane of the *z* stack, that have been categorised as adjacent (between 200 nm and 400 nm apart; Fig. S1) due to the low *z* dimension resolution afforded by conventional wide-field microscopy, are indeed colocalised in ZPA cells. At both temporal stages, the anterior/posterior differences in *Shh*/ZRS colocalisation frequency were highly significant (E10.5: *P*=0.0002; E11.5: *P*=0.0001). Owing to variation in fluorescent probe signal strength between alleles in the same nucleus, and the limited number of *z*-stack planes imaged per nucleus by SIM to minimise fluorochrome bleaching, generally less than half of all probe pairs measured from each tissue in Fig. 2 are from both alleles of the same nucleus. However, for cells in which both alleles could be measured, ZRS/*Shh* colocalisation at both occurred in 33% (E10.5) and 59% (E11.5) of ZPA cells. The proportion of ZPA cells with only one colocalised allele was 56% (E10.5) and 31% (E11.5). Only around a tenth of ZPA cells at both temporal stages had no colocalising alleles, compared with a third of distal anterior cells. By comparing conventional and SIM data for the *Shh*/ZRS probe pair in anterior and posterior tissues at two developmental stages, we show that median inter-probe distances in distal anterior limb tissues are very similar when measured by either technique (conventional=250 nm; SIM: E10.5=246 nm, E11.5=275 nm) whereas these are significantly different for ZPA cells (E10.5: conventional=221 nm, SIM=160 nm, *P*=0.01; E11.5: conventional=241 nm, SIM=136 nm, *P*<0.0001) (Fig. 2C; Tables S4, S5).

### The *Shh*/ZRS regulatory domain is compact in expressing and non-expressing tissue

**Fig. 3.**
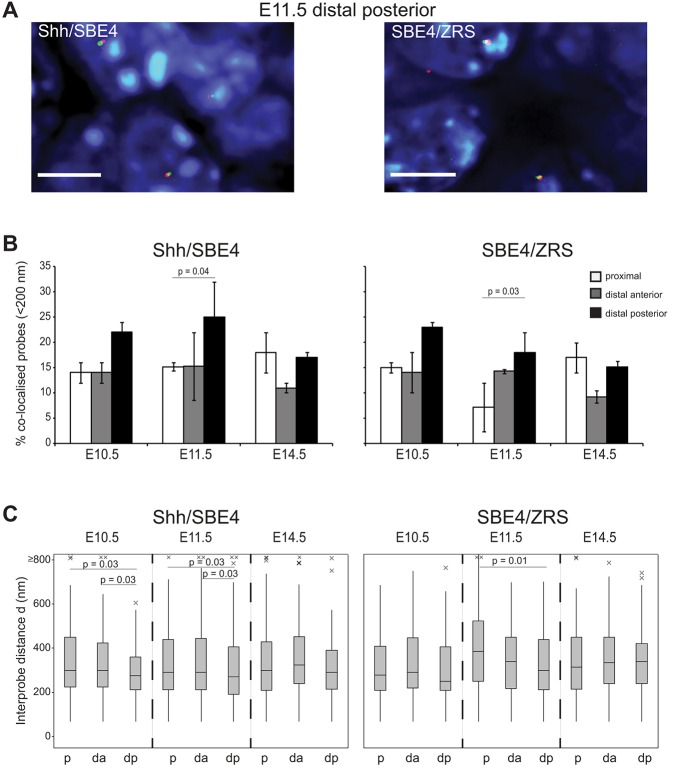
**The *Shh*-ZRS regulatory domain is maintained in a compact chromatin conformation in expressing and non-expressing tissue.** (A) Images of representative nuclei from E11.5 ZPA tissue showing FISH signals for *Shh*/SBE4 and SBE4/ZRS probe pairs. Scale bars: 5 μm. (B) Comparison of the proportion of colocalised *Shh*/SBE4 and SBE4/ZRS probe pairs (<200 nm) across the three temporal developmental stages for proximal and distal anterior and posterior (ZPA in E10.5 and E11.5 sections) forelimb tissue (*n=*70-100 alleles). Error bars represent s.e.m. obtained from two or three different tissue sections from one or two embryos. The statistical significance between data sets was examined by Fisher's exact tests. (C) Boxplots showing the distribution of interprobe distances (d) in nanometres between *Shh*/SBE4 and SBE4/ZRS in E10.5, E11.5 and E14.5 proximal (p), distal anterior (da) and posterior (dp) forelimb. The statistical significance between data sets was examined by Mann–Whitney U tests.

Long-range gene/enhancer colocalisation is often depicted as a looping out of the intervening chromatin fibre (Williamson et al., 2011). Our previous work on the *HoxD* locus implicated a gross compaction of the regulatory region, rather than a simple loop with extrusion of the intervening chromatin, upon activation of *Hoxd13* by the long-range (∼250-kb) limb-specific GCR enhancer (Williamson et al., 2012, 2014). We therefore used 3D-FISH and conventional wide-field deconvolution microscopy to measure the spatial distances between either *Shh* or the ZRS, and the SBE4 enhancer that drives *Shh* expression in the forebrain (Fig. 3A) (Jeong et al., 2006). SBE4 is located midway through the gene desert separating *Shh* and ZRS (Fig. 1A). If the entire genomic region between the gene and the limb enhancer forms a loop then *Shh*-SBE4 and SBE4-ZRS distances should be greater than those between *Shh* and ZRS.

At both temporal stages (E10.5 and E11.5) when *Shh* is active in the distal posterior limb mesenchyme, but not at E14.5, *Shh* is closer to SBE4, and *Shh*/SBE4 colocalisation frequencies are higher, compared with the other tissues analysed (Fig. 3B,C; Fig. S2A,B; Table S5). These data suggest that the genomic region between *Shh* and the ZRS is folded into a compact chromatin domain, which is at its most compact in distal posterior *Shh*-expressing cells. However, it is also apparent that the spatial distances between *Shh* and the ZRS are less than those between either *Shh*-SBE4 or SBE4-ZRS in most expressing and non-expressing tissues (Fig. S2C). These differences are significant for most tissues analysed and, intriguingly, are particularly apparent at E14.5, well past the stage of limb-specific *Shh* activity and therefore could be indicative of a constitutive chromatin conformation.

### Topography of the *Shh* regulatory domain is maintained throughout the E11.5 embryo

**Fig. 4.**
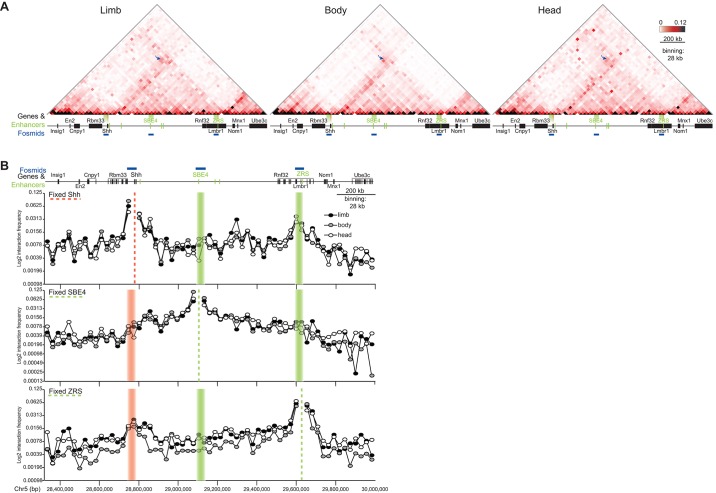
**5C-seq identifies enriched interactions between *Shh* and ZRS in E11.5 embryos.** (A) Heat maps showing 5C data from cells of the limbs, bodies and heads of E11.5 embryos, across the 1.7-Mb *Shh* region shown in Fig. 1. Heat-map intensities represent the average of interaction frequency for each window, colour-coded according to the scale shown. Interaction frequencies were normalised based on the total number of sequence reads in the 5C data set and the data shown is binned over 28-kb windows. Arrows indicate interaction frequencies between windows containing *Shh* and ZRS. Data for biological replicates are in Fig. S3A and unprocessed normalised data are shown in Fig. S4. (B) Virtual 4C analysis obtained by extracting 5C interactions with viewpoints fixed at *Shh*, SBE4 and ZRS. Dashed lines indicate the position of the fixed viewpoint from the *Shh* genomic region (pink) or regulatory elements (green). Data from limbs are in black filled circles, bodies in grey filled circles and heads in unfilled circles. Genome coordinates on chromosome 5 (Chr5) are from the mm9 assembly of the mouse genome.

Using FISH we could only infer the conformation of the *Shh* regulatory domain from the spatial relationships of three genomic loci across the *Shh*-ZRS region. To gain a more complete view of the locus, we used 5C to determine the frequency of cross-linked interactions captured between sequences in the ∼1.7 Mb region from *Insig1* ∼400 kb 3′ of *Shh* to *Ube3c* ∼350 kb beyond the ZRS (Fig. 1A) in dissected whole fore- and hindlimb buds from ∼70 E11.5 embryos (two biological replicates) (Fig. 4A, left-hand heat map; Fig. S3A; Fig. S4). We were unable to dissect cells suitable for 5C specifically from the ZPA. Three interaction domains can be identified, with the middle topologically associated domain (TAD) containing *Shh* and its entire known regulatory elements with the boundaries located 3′ of *Rbm33* and within the 5′ end of *Lmbr1*. This *Shh* regulatory TAD corresponds well with that identified by Hi-C in mouse embryonic stem cells (Dixon et al., 2012).

In limb cells, 5C cross-linked interactions are enriched between genomic fragments across the *Shh* and ZRS loci (Fig. 4A, left-hand heat map; Fig. S3A; Fig. S4). The general spatial proximity of *Shh* and the ZRS detected by FISH and inferred from enriched 5C interaction frequencies in expressing and non-expressing tissues suggests that this conformation is constitutive. To determine whether the high cross-linking efficiency of *Shh* and ZRS identified in E11.5 limb buds can also be detected in tissues where the ZRS is not active we carried out 5C on cells derived from the bodies and heads of E11.5 embryos. Even with the vast majority of these cells not expressing *Shh*, high read frequencies between *Shh* and ZRS were captured (Fig. 4A, middle and right-hand heat maps; Fig. S4) and the same TAD structures could be discerned as seen in limb tissue.

To examine more closely the regions probed by FISH (*Shh*, SBE4 and ZRS) we generated ‘virtual 4C’ plots from the 5C data (Fig. 4B; Fig. S3B) (Williamson et al., 2014). From the viewpoint of *Shh*, overall interaction frequencies with the rest of its regulatory domain are similar in limb-, body- and head-derived tissues, and are not substantially higher than those extending into the adjacent TAD 3′ of *Shh* (Fig. 4B compare the top track with the track that profiles SBE4 located in the middle of a TAD). Highest interaction frequencies for *Shh*, apart from genomic regions immediately adjacent, are with regions within the neighbourhood of ZRS (limb-specific high interactions with a loci within the gene desert that does not contain any known regulatory elements was not identified in the limb replicate data; Fig. S3B). ZRS has reciprocal enriched interactions with the *Shh* region (Fig. 4B, bottom track). However, these are not detectably higher in limb than in the embryonic body or head.

## DISCUSSION

### Activation of *Shh* in the limb bud is accompanied by colocalisation with the ZRS

Using 3D-FISH and super-resolution imaging, we provide compelling evidence that colocalisation (<200 nm) of *Shh* and the ZRS enhancer is associated with *Shh* expression in the ZPA region of the distal posterior forelimb bud, to an extent not seen in control tissues, including the limb bud after *Shh* expression has ceased at E14.5 (Figs 1, 2). The colocalisation frequencies detected by super-resolution microscopy rise to almost 80% at E11.5, suggesting that the vast majority of *Shh* alleles in the ZPA are juxtaposed to the ZRS located 1 Mb of genomic distance away. Analysis of the FISH images by either conventional wide-field or structured illumination microscopy showed a significantly higher gene-enhancer colocalisation frequency in the ZPA than in nuclei from the distal anterior region of the same limb buds (Figs 1, 2). This anterior-posterior difference in chromatin folding is consistent with our previous analysis for *Shh* and ZRS in E11.5 fore- and hindlimbs (Lettice et al., 2014) and is similar to the preferential colocalisation of *Hoxd13* and GCR in E10.5 distal posterior limb buds (Williamson et al., 2012). Like *Shh*, *Hoxd13* expression is restricted to the posterior margin of the distal limb bud at this stage. These data, however, contradict previously published work that could not identify significant differences in *Shh*-ZRS proximity between the *Shh*-expressing ZPA and distal anterior cells (Amano et al., 2009). Those data were derived from single-cell suspensions of dissected tissue from specific points across the distal limb bud whereas our data are from sections cut through whole embryos; therefore, cell/tissue preparation may be a factor in discrepancies between the data sets.

### *Shh* and its regulatory elements are located within a compact chromatin domain

Long-range interactions between genes and *cis*-regulatory elements are usually described as loops, which should be visualised as a coming together of the two loci to the exclusion of the intervening chromatin (Williamson et al., 2011; Fraser et al., 2015). Indeed a looping mechanism in distal limb could be inferred from the shorter inter-probe distances between *Shh* and ZRS, than for either of these probes with the forebrain SBE4 enhancer – even though the latter is located midway between *Shh* and ZRS on the linear chromosome (Fig. S2C). To our knowledge, this apparent *Shh*/ZRS chromatin ‘loop’ is the first to be identified by FISH.

However, the *Shh*-ZRS distances are shorter than distances to SBE4 not only in the ZPA but also in anterior limb and in E14.5 tissues when the ZRS is no longer active. But, *Shh*/ZRS colocalisation frequencies are not significant in those tissues. Another interpretation of these data is that the *Shh* regulatory domain (Fig. 4A; Fig. S3A; Fig. S4) is maintained in a tightly folded chromatin conformation where *Shh* and the ZRS are generally proximal in nuclear space. That the *Shh*-containing TAD is indeed compact can be discerned from the frequency distribution graphs, which show that most *Shh*/ZRS, *Shh*/SBE4 and SBE4/ZRS probe pairs are adjacent (200-400 nm) or colocalised (<200 nm), with median interprobe distances of between 220 and 345 nm for most tissues and developmental stages analysed (Fig. 1B; Fig. S2A,B; Table S5). This is consistent with our 5C analysis of E11.5 limb bud, body and head cells which suggests that the *Shh* regulatory region forms a constitutive self-interacting domain; the *Shh* TAD has also been identified in embryonic stem cells by Hi-C (Dixon et al., 2012). These data show somewhat enriched interactions between cross-linked DNA fragments from the genomic regions containing *Shh* and ZRS (Fig. 4; Figs S3, S4), but in all analysed tissues/cell types. The very high colocalisation frequencies that we see by microscopy between ZRS and *Shh* in the distal posterior limb at stages of *Shh* expression are not reflected in elevated interactions captured by 5C. Similarly, the increased compaction of the intervening genomic region in ZPA cells inferred from FISH analysis of distances to the neural SBE4 enhancer could not be identified by 5C. We do not know whether this is because the *Shh*-expressing (ZPA) cells do not present at a high enough proportion of cells in the dissected limb buds, or because the spatial proximities of *Shh* and ZRS, and the ZPA-specific chromatin domain, is not well captured by chromosome conformation methods (Belmont, 2014). Conversely, our previous analysis comparing 5C and FISH has highlighted that spatial proximity should not always be inferred from enriched cross-linked interactions between 3C fragments (Williamson et al., 2014).

### Facilitating gene regulation by enhancer–promoter proximity

Here, we have shown that local chromatin conformation maintains spatial proximity of *Shh* with the regulatory domain containing its enhancers – including the limb-specific enhancer ZRS – in a variety of cell types, not just those expressing *Shh*. If the physical interaction of active enhancers and their target gene promoters is essentially a stochastic process, their constitutive relative proximity within the same chromatin domain could be advantageous – for example, by reducing the search space of the enhancer for the promoter (Williamson et al., 2011; Benabdallah and Bickmore, 2015). Consistent with this model, we have previously shown that, in the limb, expression levels of a reporter gene inserted into several positions across the whole *Shh* regulatory domain, is highest when the reporter inserts close to either the ZRS or *Shh* compared with insertion sites within the intervening gene desert (Anderson et al., 2014). These data suggest that ZRS-induced expression requires direct or indirect interactions with the target gene and these interactions are optimised by minimising the search space within a constrained chromatin domain. Whether the actual colocalisation of the ZRS with *Shh* in the ZPA is a cause or consequence of limb-specific *Shh* activation remains to be determined.

## MATERIALS AND METHODS

### Fish

For 3D-FISH, E10.5, E11.5 and E14.5 embryos from CD1 mice were collected, fixed, embedded, sectioned and processed as previously described (Morey et al., 2007), except that sections were cut at 6 μm. Fosmid clones (Fig. 1A; Table S1) were prepared and labelled as previously described (Morey et al., 2007). Between 160 and 240 ng of biotin- and digoxigenin-labelled fosmid probes were used per slide, with 16-24 μg of mouse Cot-1 DNA (Invitrogen) and 10 μg salmon sperm DNA.

### Image analysis

For 3D analysis of tissue sections by conventional microscopy, slides were imaged with a Hamamatsu Orca AG CCD camera (Hamamatsu Photonics), Zeiss Axioplan II fluorescence microscope with Plan-neofluar or Plan apochromat objectives, a Lumen 200 W metal halide light source (Prior Scientific Instruments) and Chroma #89014ET single excitation and emission filters (Chroma Technology Corporation) with the excitation and emission filters installed in Prior motorised filter wheels. A piezoelectrically driven objective mount (PIFOC model P-721, Physik Instrumente & Co, Karlsruhe) was used to control movement in the *z* dimension. Hardware control, image capture and analysis were performed using Volocity (PerkinElmer). Images were deconvolved using a calculated point spread function with the constrained iterative algorithm of Volocity (PerkinElmer). Image analysis was carried out using the Quantitation module of Volocity (PerkinElmer).

### SIM imaging

Images were acquired using structured illumination microscopy (SIM) performed on an Eclipse Ti inverted microscope equipped with a Nikon Plan Apo TIRF objective (NA 1.49, oil immersion) and an Andor DU-897X-5254 camera. Laser lines at 405, 488 and 561 nm were used. Step size for *z*-stacks was set to 0.120 μm, which is well within the Nyquist criterion. For each focal plane, 15 images (five phases, three angles) were captured with the NIS-Elements software. SIM image processing and reconstruction were carried out using the N-SIM module of the NIS-Element Advanced Research software. Image analysis was carried out using the Quantitation module of Volocity (PerkinElmer) with *x* and *y* binning resolution of 32 nm.

### 3C library preparation

Limbs from ∼70 E11.5 embryos, three E11.5 embryos with the limbs and heads removed, and the heads of three E11.5 embryos were collected in 15 ml tubes with enough PBS to cover them and to dissociate the cells by repeated pipetting with enlarged tip ends. Cells were fixed with 1% formaldehyde for 10 min at room temperature (r.t.). Crosslinking was stopped with 125 mM glycine, for 5 min at r.t. followed by 15 min on ice. Cells were centrifuged at 400 ***g*** for 10 min at 4°C, supernatants removed and cell pellets flash frozen on dry ice.

Cell pellets were treated as previously described (Dostie and Dekker, 2007; Ferraiuolo et al., 2010; Williamson et al., 2014). *Hin*dIII-HF (NEB) was the restriction enzyme used to digest the crosslinked DNA.

### 5C primer and library design

5C primers covering the *Usp22* (mm9, chr11: 60,917,307-61,003,268) and *Shh* (mm9, chr5: 28,317,087-30,005,000) regions were designed using ‘my5C.primer’ (Lajoie et al., 2009) and the following parameters: optimal primer length of 30 nt, optimal TM of 65°C, default primer quality parameters (mer:800, U-blast:3, S-blasr:50). Primers were not designed for large (>20 kb) and small (<100 bp) restriction fragments, for low complexity and repetitive sequences, or where there were sequence matches to >1 genomic target. The *Usp22* region was used to assess the success of each 5C experiment but was not used for further data normalisation or quantification.

The universal A-key [CCATCTCATCCCTGCGTGTCTCCGACTCAG-(5C-specific)] and the P1-key tails [(5C-specific)-ATCACCGACTGCCCATAGAGAGG] were added to the forward and reverse 5C primers, respectively. Reverse 5C primers were phosphorylated at their 5′ ends. An alternating design consisting of 365 primers in the *Shh* region (182 forward and 183 reverse primers) was used. Primer sequences are listed in Table S6.

### 5C library preparation

5C libraries were prepared and amplified with the A-key and P1-key primers as previously described (Fraser et al., 2012). Briefly, 3C libraries were first titrated by PCR for quality control (single band, absence of primer dimers, etc.), and to verify that contacts were amplified at frequencies similar to those usually obtained from comparable libraries (same DNA amount from the same species and karyotype) (Dostie and Dekker, 2007; Dostie, et al., 2007; Fraser, et al., 2010). We used 1-10 μg of 3C library per 5C ligation reaction.

5C primer stocks (20 μM) were diluted individually in water on ice, and mixed to a final concentration of 2 nM. Mixed diluted primers (1.7 μl) were combined with 1 μl of annealing buffer (10× NEBuffer 4, New England Biolabs) on ice in reaction tubes. To each tube 1.5 μg salmon testis DNA was added, followed by the 3C libraries and water to a final volume of 10 μl. Samples were denatured at 95°C for 5 min, and annealed at 55°C for 16 h. Ligation with Taq DNA ligase (10 U) was performed at 55°C for one hour. One tenth (3 μl) of each ligation was then PCR amplified individually with primers against the A-key and P1-key primer tails. We used 26 cycles based on dilution series showing linear PCR amplification within that cycle range. The products from three to five PCR reactions were pooled before purifying the DNA on MinElute columns (Qiagen).

5C libraries were quantified by bioanalyser (Agilent) and diluted to 26 pmol (for Ion PGM Sequencing 200 Kit v2.0). One microlitre of diluted 5C library was used for sequencing with an Ion PGM Sequencer. Samples were sequenced onto Ion 316 Chips following the Ion PGM Sequencing 200 Kit v2.0 protocols as recommended by the manufacturer (Life Technologies).

### 5C data analysis

Analysis of the 5C sequencing data was performed as previously described (Berlivet et al., 2013). Sequencing data were processed through a Torrent 5C data transformation pipeline on Galaxy (https://main.g2.bx.psu.edu/). Data were normalised by dividing the number of reads of each 5C contact by the total number of reads from the corresponding sequence run. All scales shown correspond to this ratio multiplied by 10^3^. For each experiment, the number of total reads, and of used reads, is provided in Table S7. The unprocessed heat maps of the normalised 5C datasets can be found in Fig. S4.
